# Research progress on emerging and important Tick-Borne pathogens

**DOI:** 10.3389/fmicb.2026.1866307

**Published:** 2026-06-10

**Authors:** Gang Duan, Linghan Kong, Sicheng Duan, Shujun Nie, Wanjiang Gu

**Affiliations:** 1 Chongqing Key Laboratory of Highly Pathogenic Microbes, Chongqing Research Center for Disease Prevention and Public Health, Chongqing Center for Disease Control and Prevention, Chongqing Academy of Preventive Medicine, Chongqing, China; 2 Chongqing Wanzhou District Center for Disease Control and Prevention, Chongqing, China; 3 College of Humanities and Sciences, National University of Singapore, Singapore, Singapore

**Keywords:** *Anaplasma*, *Babesia*, *Borrelia*, emerging pathogens, *Rickettsia*, SFTSV, TBEV, Tick-borne diseases

## Abstract

Ticks are important vector arthropods, which can carry and transmit a variety of pathogenic microorganisms, and pose a serious threat to global public health. This study reviews the research progress of the main and emerging tick-borne pathogens, such as Lyme disease related *Borrelia, Rickettsia, Babesia*, Thrombocytopenia Syndrome Virus (SFTSV), Tick-borne Encephalitis Virus (TBEV), Alongshan virus (ALSV), etc., focuses on their genomic diversity, pathogenicity, transmission and immune escape, co- infection. In addition, the application of new detection technology [Clustered Regularly Interspaced Short Palindromic Repeats (CRISPR), metagenomic next-generation sequencing (mNGS), microfluidics] in Tick-Borne pathogens is summarized.It highlights current research limitations, including delayed vaccine development and inadequate surveillance systems. Finally, future research directions are prospected, providing theoretical references for the prevention and control of tick-borne diseases.

## 1 Introduction

Ticks, as obligate blood-sucking arthropods, can invade a wide range of vertebrate hosts and are the vector of a large number of pathogens. As the second largest vector of human infectious diseases after mosquitoes, Ticks are also the main vector for pathogen transmission between livestock and wild animals. Ticks carry a variety of pathogenic pathogens, which can cause Lyme disease, tick-borne encephalitis, *Rickettsia* disease and other diseases. Some of these diseases have a high mortality, which can lead to serious complications and even death. Such diseases not only pose a serious threat to human health, but also seriously hinder the development of animal husbandry and cause a heavy social and economic burden (De la Fuente et al., 2008; Boulanger et al., 2019).

Under the influence of global climate change and human activities, the epidemic pattern of tick-Borne diseases has changed significantly. Global warming has promoted the continuous expansion of tick habitats. Tick species that were originally only distributed in specific areas have achieved cross-regional diffusion by means of migratory birds migration, livestock and poultry transportation, etc., colonizing the new ecological environment and forming effective vector populations (Backus et al., 2022; Narvaez et al., 2024; Chitimia-Dobler et al., 2018; Ning et al., 2017; Drehmann et al., 2019). The development and utilization of the natural environment and the increase of outdoor leisure activities further improve the contact probability between people and ticks and epidemic focus. In addition, pathogenic microorganisms continue to undergo gene recombination and mutation in the process of evolution, and new Tick-Borne pathogens continue to be found. Some new pathogens have strong pathogenicity and transmission ability, which aggravates the risk of outbreak of Tick-Borne diseases, and puts forward higher requirements for prevention and control.

Although existing studies have initially revealed the diversity and transmission characteristics of Tick-Borne pathogens, most studies focus on a single pathogen or local area, and lack of integrated analysis of pathogen spectrum at the global scale; Molecular analyses of pathogen–vector–host interactions remain superficial, which is difficult to support the accurate prediction of cross regional transmission risk; At the same time, the transformation efficiency of basic research results into prevention and control technologies such as diagnosis, vaccines and drugs is low, and there is a significant gap with the actual prevention and control needs. Based on this, combined with the research results of Tick-Borne pathogens at home and abroad in recent years, this paper systematically summarizes the diversity, evolution and transmission characteristics of major Tick-Borne pathogens, analyzes the research status of new Tick-Borne pathogens, and points out the deficiencies and controversial points of existing research, so as to provide theoretical support and improvement direction for Tick-Borne disease prevention and control and related scientific research work.

## 2 Methods

This study systematically searched PubMed database and collected the original literatures and reviews related to tick-borne diseases published in English before April 21, 2026. The search terms of major Tick-Borne pathogens were: (Tick-Borne pathogens OR Borrelia OR Rickettsia OR Anaplasma OR Babesia OR SFTS virus OR Tick-Borne Encephalitis Virus) AND (molecular mechanism OR pathogenesis OR genome). For emerging tick-borne pathogens, the search terms were: (emerging tick-borne pathogens OR novel tick-borne virus OR new Tick-Borne bacteria) AND (Ticks OR Ixodidae). Detection technologies were searched using: tick-borne disease AND microfluidics, tick-borne disease AND metagenomic next-generation sequencing, and tick-borne disease AND Clustered Regularly Interspaced Short Palindromic Repeats (CRISPR) AND diagnosis. Inclusion criteria: English literature, covering the diversity, evolution, pathogenesis, detection technology and control progress of Tick-Borne pathogens. Exclusion criteria: letters, comments, short communications, non-English literature and unrelated literature. All articles were imported into EndNote for management, yielding 2,036 records. After removing duplicates and preliminary screening by title and abstract, 261 articles underwent full-text evaluation, with 133 ultimately included for this review.

## 3 Diversity, evolution, and research advances of major Tick-Borne pathogens

### 3.1 Lyme disease-related *Borrelia*

*Borrelia burgdorferi* sensu lato complex represent the most prevalent Tick-Borne pathogenic group in temperate regions of the Northern Hemisphere. Their evolution and transmission are governed by multiple factors, including species divergence, vector distribution, host-pathogen interactions, and environmental cues, rendering this pathogen group a key focus of global public health concern (Mead, 2022). Pronounced intercontinental differentiation exists in the species diversity of this complex: *Borrelia burgdorferi* sensu stricto dominates in North America, while *Borrelia afzelii* and *Borrelia garinii* are the predominant species in Europe, with pathogenic species such as *Borrelia miyamotoi* also detected in certain regions. This heterogeneity directly contributes to distinct clinical manifestations of Lyme disease between Europe and North America (Marques et al., 2021; Gray et al., 2024; Cialini et al., 2025). In parts of Asia (South Korea), CO infection transmission of Borrelia burgdorferi and Anaplasma phagocytophilium has been confirmed (Ma et al., 2024).

Whole-genome analysis of 47 global isolates reveals a clonal structure of the core *Borrelia* genome, whereas plasmid-encoded lipoprotein genes involved in host interactions represent recombination hotspots. Natural selection and genetic recombination jointly drive genomic diversification, laying the molecular foundation for cross-host adaptation (Akther et al., 2024). Strain diversity profoundly impacts pathogenicity and tissue tropism: different strains of *Borrelia burgdorferi* sensu stricto exhibit marked variations in cardiac invasiveness, *Borrelia garinii* is more prone to inducing neuroborreliosis, and *Borrelia afzelii* is frequently associated with cutaneous and articular lesions (Pfeifle et al., 2025; Baux et al., 2025). During transmission, *Borrelia* must breach the sequential barriers of the tick midgut, hemocoel, and salivary glands, as well as host immune defenses. Stage-specific expression of outer surface lipoproteins including OspA, OspB, and OspC plays a pivotal role in cross-host adaptation, immune evasion, and tissue colonization (Rana et al., 2023; Radolf et al., 2021; O'Bier et al., 2024).

The geographical distribution of vector tick species directly dictates the spread range of the pathogen, with *Ixodes scapularis* in North America and *Ixodes ricinus* in Europe serving as the primary transmission vectors (Gray et al., 2024; Couret et al., 2022). The infection rate of adult *Ixodes scapularis* ticks in northeastern North America reaches 49%–54% and continues to rise, while the infection rate of *Ixodes ricinus* ticks in southern Sweden is as high as 35% (Cialini et al., 2025; Price et al., 2024). Geographic expansion of tick vectors, closely linked to global warming and habitat alterations, has further broadened the transmission range of *Borrelia* (Wimms et al., 2023; Socarras et al., 2022).

Host communities exert dual regulatory effects on the transmission cycle: wildlife migration facilitates cross-regional pathogen dispersal, whereas hosts such as roe deer and fallow deer can eliminate *Borrelia* via serum activity, generating a dilution effect. Small mammals including *Apodemu*s and voles act as the main reservoir hosts, with their population density directly modulating transmission efficiency (Cialini et al., 2025; Veinović et al., 2024). Human genetic background also influences susceptibility to infection: the P53L missense mutation in the *SCGB1D2* gene impairs inhibitory activity against *Borrelia*, significantly elevating infection risk (Strausz et al., 2024). Furthermore, *Borrelia* often causes coinfections with *Anaplasma phagocytophilum* and *Babesia* species, which may exacerbate disease severity and complicate clinical diagnosis and treatment (Ma et al., 2024; Veinović et al., 2024).

Lyme disease typically presents with erythema migrans as an early hallmark; untreated cases can progress to multi-system damage involving the nervous system, joints, heart, and other organs (Baux et al., 2025). Conventional two-tiered serological testing suffers from low sensitivity in the early stage of infection, and improved algorithms coupled with novel molecular diagnostic techniques are gradually optimizing diagnostic efficacy (Guérin et al., 2023; Taylor-Salmon and Shapiro, 2024). Early infections respond favorably to antibiotics such as doxycycline, yet effective interventions for chronic infections remain lacking, with lactate dehydrogenase identified as a potential therapeutic target (Sze et al., 2025). Multivalent chimeric vaccines based on OspA and OspC exhibit promising prospects, with protective efficacy reaching 80%–90% (O'Bier et al., 2024).

Genomic analyses of Borrelia are often limited by restricted isolate collections and short-term sampling strategies, which impair the ability to resolve fine-scale evolutionary trajectories and transmission routes. While preclinical data support the potential of vaccine candidates, these formulations have not been evaluated in large-scale clinical trials, with critical gaps remaining in the assessment of cross-genotype protection and long-term safety. Mechanistic inquiries into chronic Lyme disease are largely correlational, lacking definitive *in vivo* validation to substantiate their clinical relevance and translational potential. Improvements in diagnostic approaches have been modest rather than paradigm-shifting, and point-of-care diagnostic modalities remain underutilized in endemic regions, thereby hindering prompt case identification and clinical management.

### 3.2 Rickettsia

*Rickettsia* are obligate intracellular Gram-negative bacteria, and spotted fever group *Rickettsia* (SFGR) represents the predominant pathogenic lineage within this genus. Their evolution and transmission rely on synergistic interplay between tick vectors and vertebrate hosts, characterized by distinct geographical specificity and strong adaptability to tick vectors (Kim, 2022). To date, 48 SFGR species have been identified globally, transmitted by 146 tick species and classified into five ecological clusters; among them, *Rickettsia felis* and *Rickettsia conorii* pose pervasive threats to human public health (Zhang et al., 2023b).

Obvious heterogeneity exists in species diversity and pathogenicity across regions: *Ixodes ricinus* and *Rhipicephalus* ticks serve as the primary transmission vectors. *Rickettsia rickettsii* in the Americas can induce fatal Rocky Mountain spotted fever, whereas *Rickettsia parkeri* gives rise to mild spotted fever (Rodrigues et al., 2023; Martiniano et al., 2022). The overall prevalence of SFGR in ticks collected from Ningxia, China reaches 49.4%, accompanied by the identification of a novel candidate SFGR species (Zhu et al., 2025).

As for the immune escape mechanism, *Rickettsia* species use specific virulence effectors to manipulate host cells. For example, Rickettsia's outer membrane protein B (OmpB) can interact with host receptor Ku70 to mediate cell invasion, while other effectors (such as Sec7) help bacterial growth by inhibiting phagosome lysosome fusion (Rana et al., 2023). Unlike the pathogenic phagocytic Anaplasma, Rickettsia buchneri had little effect on the bioenergy of ticks and could not induce significant glycolysis (Samaddar et al., 2024). Climate warming and habitat transformations have facilitated the geographical expansion of *Rickettsia*. Novel epidemic strains have been documented in Kazakhstan, Iran and adjacent regions, leading to a sustained elevation in cross-regional transmission risk (Dong et al., 2023; Ghavami et al., 2024).

Studies have predominantly centered on virulence genes and surface antigens, yet the molecular pathways underlying pathogen-mediated metabolic and immune manipulation in tick vectors remain fragmented and incompletely characterized. Geographic surveillance studies frequently employ convenience sampling approaches and non-standardized PCR methodologies, which substantially compromise the comparability of findings across different research cohorts and regions. For the majority of novel or candidate Rickettsia species, formal pathogenicity assessments using animal models and epidemiological investigations linking these pathogens to human cases are lacking, resulting in unquantified public health risks. Additionally, the drivers of cross-regional pathogen spread are often oversimplified to climatic and habitat factors, with insufficient consideration of livestock movement, international trade, and human behavioral patterns—factors that collectively weaken the robustness and predictive capacity of current transmission models.

### 3.3 Anaplasma

The global infection prevalence of this bacterium in ticks is approximately 4.76%. In the northeastern United States, infection rates in adult *Ixodes scapularis* ticks range from 4% to 9%, and coinfection with *Borrelia burgdorferi*, the causative agent of Lyme disease, occurs at 8.2%. Similar to the complex strain level changes observed in its close relative Babesia, phagocytic *Anaplasma* also shows significant genetic diversity, thus affecting the potential and transmission dynamics of zoonoses (Price et al., 2024; Zintl et al., 2023; Karshima et al., 2022).

*Anaplasma capra* is an emerging species that has been reported across Asia, Europe, and Africa, with two recognized genotypes. Most human-derived isolates belong to genotype 2 (Altay et al., 2024). Establishment of *A. capra* infection in ticks requires the type IV secretion system effector AteA. This pathogen also modulates tick glycolytic metabolism to support its survival and colonization within the vector (Samaddar et al., 2024; Park et al., 2023).

The global prevalence estimates reported in studies show significant differences, which are closely related to variations in detection methods, tick developmental stages, and ecological settings, and no standardized meta-analytical framework has been established to date. For Anaplasma capra (A. capra), fundamental epidemiological data remain severely limited, including key information such as definitive vectors, reservoir hosts, human seroprevalence, and clinical spectrum, all of which remain unclear. Relevant mechanistic studies are only focused on the glycolytic pathway, while the specific effects of this pathogen on lipid metabolism, amino acid metabolism, and redox metabolism have not yet been explored. In addition, research on the synergy and clinical outcomes of co-infection in humans is relatively weak, which to a certain extent limits the clinical diagnosis and treatment guidance for severe cases.

### 3.4 Babesia

*Babesia* are apicomplexan protozoans, with *Babesia microti* representing the principal species pathogenic to humans, transmitted primarily by specific tick vectors including *Ixodes ricinus* (castor bean tick). To date, approximately 250 *Babesia* species have been described worldwide, with host associations covering 73 tick vector species and 224 vertebrate species (Fu et al., 2025; Bajer and Dwuznik-Szarek, 2021). Unlike the highly zoonotic *B. microti* sensu stricto, the “Munich” strain identified in Ireland is associated with limited public health risk (Zintl et al., 2023); Studies have shown that the activation of tick Toll pathway can induce the expression of defensin, which plays a key role in controlling *Babesia* minimus infection in the vector (Jalovecka et al., 2024). *Babesia* pathogens commonly establish coinfections with *Rickettsia* and *Anaplasma* species, the presence of these pathogens usually triggers up regulation of tick *defensin* gene expression (Szczotko et al., 2024).

Taxonomic uncertainty in studies has led to inconsistent naming of such pathogens and overestimated species diversity calculations, which affects the accuracy and comparability of research data. Mechanistic studies are limited to the Toll pathway, while other immune signaling modules and cellular responses have not yet been systematically characterized. The molecular basis of strain-specific virulence remains unclear, making it difficult to conduct effective risk stratification. Research related to coinfection mainly focuses on vector competence, with insufficient attention paid to clinical severity, treatment response, and long-term outcomes, thereby limiting its translational relevance.

### 3.5 Fever with Thrombocytopenia Syndrome Virus (SFTSV) and Tick-Borne Encephalitis Virus (TBEV)

Thrombocytopenia Syndrome Virus (SFTSV) and Tick-borne Encephalitis Virus (TBEV) represent the two most clinically significant tick-borne viral pathogens worldwide. Members of the *Bunyaviridae* and *Flaviviridae* families, respectively, both viruses show evolution, transmission dynamics, and genomic diversity tightly linked to host immunity, vector adaptation, and environmental shifts, establishing them as priority targets for global public health intervention (Johnson et al., 2023; Chiffi et al., 2023).

Thrombocytopenia Syndrome Virus is an emerging tick-borne bunyavirus associated with a case fatality rate of 5%–30%. Originally documented in rural and mountainous areas of East Asia, its distribution has expanded into urban settings; both the virus and its primary vector, the long-horned tick, have been detected in urban parks inside Beijing's Fifth Ring Road (Seo et al., 2021; Woo et al., 2025; Yuan et al., 2024). The virus displays extensive genomic diversity. In the Hangzhou region, five pure genotypes (A, B-2, D, E, F) and seven reassortant genotypes have been identified, with genotype E significantly associated with higher case fatality rates. Molecular dating suggests viral divergence began around 1,785, with subsequent dispersal across regions mediated by migratory birds, leading to a multicentric epidemic structure (Wen et al., 2024). Beyond tick-bite transmission, animal-to-human and human-to-human routes have been confirmed. Exposure to dogs and contact with infectious bodily fluids substantially elevate infection risk, and occasional family clusters have been reported (Woo et al., 2025).

At the pathogenic level, SFTSV enters host cells through its Gn/Gc glycoproteins, while the non-structural protein NSs suppresses host innate immune signaling. The virus replicates in platelets and stimulates hyperactivation, triggering coagulation disorders that culminate in thrombocytopenia and multiple organ failure (Sun et al., 2025; Fang et al., 2023). N6-methyladenosine (m6A) RNA modification critically supports infection: the viral nucleoprotein sequesters host m6A regulatory factors to enhance mRNA translation and genome stability. This modification is highly conserved in tick cells, providing a molecular mechanism for cross-species transmission (Chen et al., 2024c). The host factor IFITM3 binds the viral Gc protein to block cellular entry, while *Akkermansia*-derived hamalin mitigates systemic inflammation, highlighting novel therapeutic targets (Du et al., 2024; Xie et al., 2023). Currently, there are no licensed vaccines for SFTSV, and clinical care remains highly supportive. Although neutralizing antibodies and favipiravir show preliminary promise, large-scale clinical validation is still lacking (Zhang et al., 2025; Yu et al., 2026).

Tick-borne Encephalitis Virus is the dominant tick-borne encephalitic virus across Eurasia, with a geographic range that continues to expand. Endemic in many European countries and Heilongjiang Province, China, its spread is driven primarily by climate warming (Chiffi et al., 2023; Chen et al., 2024a). In addition to transmission by tick bite, food-borne infection has emerged as an important secondary route; consumption of unpasteurized cow's or goat's milk can cause household clusters, with children at elevated risk (Ličková et al., 2021; Buczek et al., 2022). TBEV is a positive-sense single-stranded RNA virus encoding three structural and seven non-structural proteins. The envelope (E) protein mediates viral attachment and membrane fusion, whereas NS5 interacts with SIRT1 to inhibit DNA damage repair, thereby worsening neurological injury (Pulkkinen et al., 2022; Sui et al., 2024). LRP8 has been identified as a key receptor for central nervous system entry; soluble receptor decoys targeting LRP8 effectively neutralize the virus, supporting a novel strategy for antiviral development (Li et al., 2025).

Tick-borne Encephalitis Virus infection typically follows a biphasic clinical course, and 40%–50% of patients develop long-term neurological sequelae. Severe illness and cognitive impairment are also well-documented in pediatric cases (Chiffi et al., 2023; Parfut et al., 2023). Distinct transcriptional responses in neurons, astrocytes, and microglia offer insights into the neuropathogenic mechanisms of TBEV (Rosendal et al., 2024). Licensed vaccines in Europe confer >92% protection and strongly reduce infection risk. However, no specific antiviral therapies are available, and nucleoside analogs and other candidates remain in preclinical or early clinical development (Angulo et al., 2023).

Transmission of both viruses displays strong seasonal and geographic patterns. Range expansion of tick vectors, host migration, viral mutation, and immune evasion together drive the growing global burden of tick-borne viral diseases, representing a central challenge for control programs. Future strategies will require strengthened surveillance, improved diagnostic platforms, and the development of next-generation vaccines and therapeutics based on newly identified molecular targets.

For SFTSV, Bird-mediated dispersal has been hypothesized, but direct tracking evidence and genomic phylogeographic data are still lacking. m6A, IFITM3, and hamalin findings are limited to *in vitro* or mouse models; human relevance is unconfirmed. Favipiravir and neutralizing antibody trials are small, non-randomized, and short–term, limiting clinical adoption. For TBEV, foodborne risk is understudied globally with limited dairy monitoring. Neurological sequelae mechanisms are poorly defined, hindering rehabilitation strategies. Antiviral development is slow, with poorly characterized off–target effects. Coinfections and interactions with other pathogens are entirely unstudied, preventing full epidemiological modeling.

### 3.6 Comparative analysis of major Tick-Borne pathogens

A comparative analysis of major Tick-Borne pathogens, ncluding their transmission cycles, pathogenic mechanisms, immune evasion strategies, and control measures is presented in Table 1.

**Table 1:** Comparative analysis of major Tick-Borne pathogens.

Pathogen	Transmission cycle	Pathogenesis	Immune evasion strategies	Control measures
Borrelia	*Ixodes*-borne; The host community has dual regulatory effects on transmission; Co infection.	Significant differences in tissue tropism among different genospecies.	stage-specific expression of OspA, OspB and OspC; plasmid-encoded lipoprotein genes serve as recombination hotspots.	Doxycycline; multivalent vaccines.
Rickettsia	*Ixodes*-borne; synergistic interactions between ticks and vertebrate hosts.	Regulate tick metabolic and immune pathways to improve transmission adaptability; Virulence effectors mediate cytoskeletal rearrangement.	Plasmid-encoded surface protein gene recombination leads to antigen variation and immune evasion;unclear molecular pathway mechanism.	vector surveillance;Ecological environment management.
Anaplasma	Studies show that the tick carriage averages 4.76%; co infections with *Borrelia*.	Type IV effector AteA mediates infection; modulate tick glycolysis for colonization.	Modulates tick metabolism to adapt to the vector and sustain transmission.	Weak research on metabolism and mixed infection.
Babesia	*Ixodes ricinus*-borne.	Pathogenicity difference of different strains.	Activates tick Toll pathway; modulates defensin expression.	Insufficient research on clinical diagnosis and treatment of mixed infection.
SFTSV	Main vector *Haemaphysalis longicornis*; animal/human-to-human spread.	Invades host cells via envelope glycoproteins Gn/Gc, causing coagulation disorders.	NSs suppresses innate immunity; m6A modification enhances replication.	Supportive treatment; favipiravir;neutralizing antibody.
TBEV	Tick bite; food-borne via unpasteurized milk.	Neuroinvasion via LRP8; neuronal injury; long-term sequelae.	Envelope-mediated escape; NS5 inhibits DNA repair.	Licensed vaccines; symptomatic treatment.

Data compiled from cited references.

### 3.7 Coinfection of Tick-Borne pathogens

In tick-borne diseases, Borrelia burgdorferi, Rickettsia, Anaplasma, Babesia and some new pathogens are often co-infected. The coexistence of multiple pathogens can significantly change the disease process and have an important impact on clinical symptoms, diagnosis and treatment (Cutler et al., 2021).

Aggravating Clinical Symptoms: Co-infection can significantly aggravate the disease, increase the severe rate and mortality. When Lyme disease is combined with Anaplasma phagocytophilum or Babesia infection, the risk of multiple system damage is significantly increased (Ma et al., 2024).

Increasing Diagnostic Difficulty: The clinical symptoms of co-infection are lack of specificity and are prone to misdiagnosis and missed diagnosis (Ma et al., 2024; Cutler et al., 2021). Traditional detection methods are mostly targeted at a single pathogen, which is difficult to diagnose mixed infection, increasing the difficulty of diagnosis (Price et al., 2024; Karshima et al., 2022; Cutler et al., 2021).

Decreased therapeutic effect: A single antibiotic is difficult to cover the mixed infection of bacteria and protozoa, and the curative effect is reduced. When bacteria and protozoa coexist, the lack of a unified treatment scheme increases the difficulty of clinical treatment (Ma et al., 2024; Sze et al., 2025; Cutler et al., 2021).

Poor prognosis: The severe rate and mortality rate of patients with co-infection are higher. The risk of chronic sequelae (nerve, joint, heart injury) is increased, and the rehabilitation cycle is prolonged (Baux et al., 2025; Parfut et al., 2023).

## 4 Emerging Tick-Borne pathogens: discovery, diversity, and public health implications

**Figure 1:**
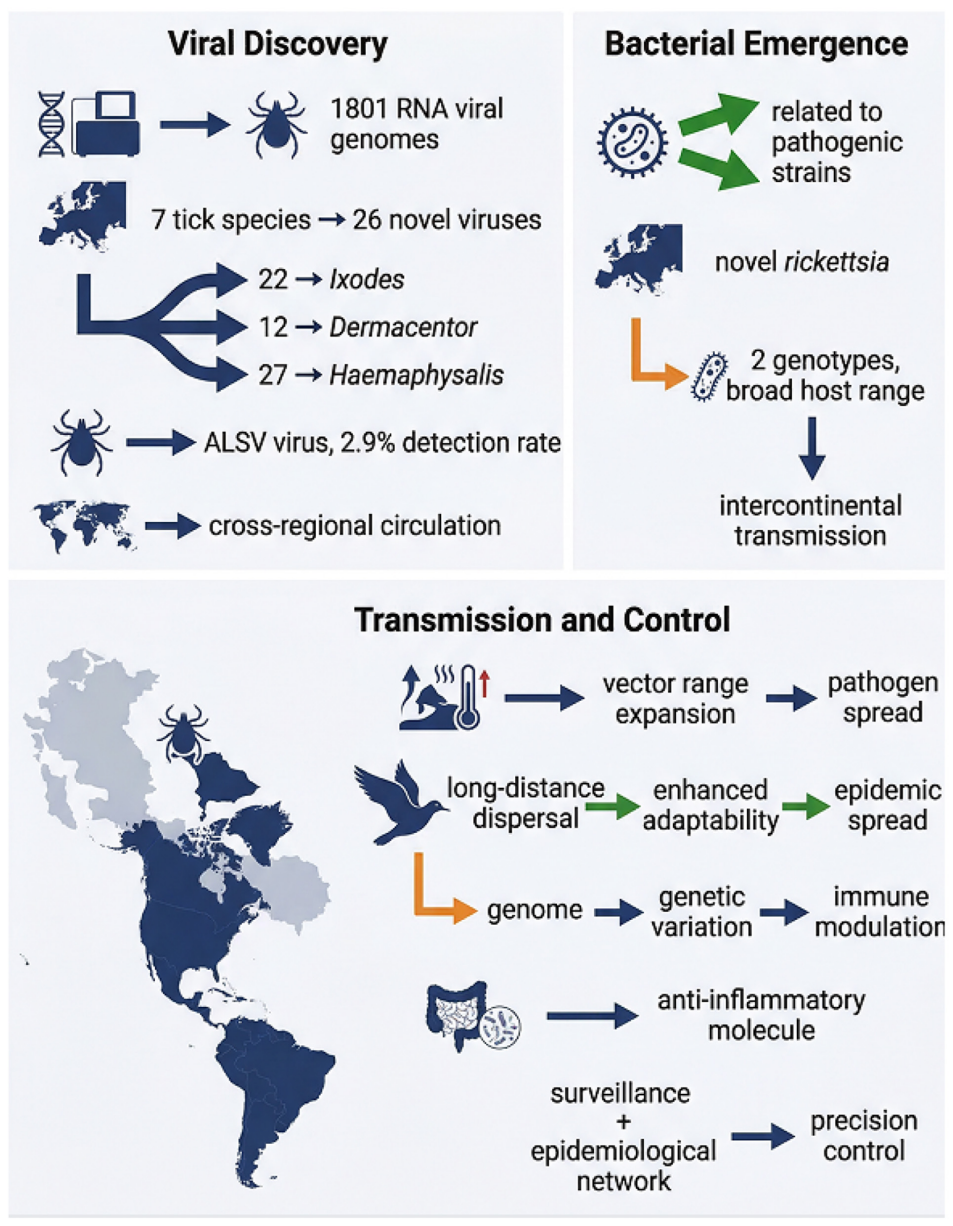
Schematic diagram of the diversity of newly emerging Tick-Borne pathogens globally and the prevention and control of their transmission. This figure was created based on information and data from previous studies (Ni et al., 2023; Tian et al., 2025; Du et al., 2025; Ergunay et al., 2024a; Gömer et al., 2024; Wang et al., 2024; Jamil et al., 2025; Altay et al., 2024; Sheng et al., 2025; Shimojima et al., 2024).

As shown in Figure 1, new Tick-Borne pathogens continue to be found. With global warming and long-distance transmission through birds, they may pose an increasingly serious threat to global public health (Figure 1).

New Tick-Borne pathogens continue to emerge and spread globally, representing a growing threat to public health. These agents span diverse viral and bacterial taxa, whose evolution and transmission are tightly associated with vector adaptation, host–vector interactions, and environmental changes, resulting in pronounced regional heterogeneity and variable pathogenic potential (Ni et al., 2023; Tian et al., 2025; Ergunay et al., 2024a; Du et al., 2025).

### 4.1 Novel tick-borne viruses

New viruses constitute the majority of novel pathogens, with species diversity far exceeding previous understanding. A global metatranscriptomic analysis of 31 tick species recovered 1,801 RNA viral genomes in a single investigation, establishing ticks as major natural reservoirs for RNA viruses (Ni et al., 2023). In Ningxia, China, 26 novel viruses were identified across seven tick species from three genera. Viral composition displayed strong genus-specificity: 22 viruses were unique to *Ixodes*, 12 to *Dermacentor*, and 27 to *Haemaphysalis*, reflecting close associations with tick geographic distribution and ecological traits (Tian et al., 2025). In Mexico, seven novel viruses were detected in ticks, including members of *Bunyavirales* and *Flaviviridae*; several are distantly related to known pathogenic viruses and may represent emerging public health threats (Laredo-Tiscareño et al., 2025).

Alongshan virus (ALSV) can antagonize the host's type I interferon response. Non-structural protein (*nsp1*) binds and degrades human *stat2* protein through autophagy, directly inhibiting the expression of interferon-stimulated gene (*ISG*). *Nsp1* can also induce mitochondrial autophagy and inhibit mitochondrial production, further weakening the host immune response. The methyltransferase of NSP can specifically bind to the key site F175/R176 in *stat2*. Inhibiting mitochondrial autophagy with 3-methyladenine or promoting mitochondrial formation with pioglitazone can reverse the inhibitory effect of *nsp1* on *ISG*, which provides a potential strategy for antiviral therapy (Zhao et al., 2024).

Among recently recognized viral pathogens, the global expansion of ALSV is especially prominent. ALSV is a tetrapartite, single-stranded positive-sense RNA virus with surface glycoproteins carrying Asian lineage-specific signatures. Transmitted by *Ixodes persulcatus*, this virus was detected in 2.9% of tick samples from Mongolia, and human infections have been documented across multiple Asian and European countries (Du et al., 2025; Gömer et al., 2024). ALSV was identified for the first time in tick surveys from Poland; its genome falls within the European lineage with no evidence of recombination, indicating sustained endemic circulation via tick-borne transmission (Ergunay et al., 2025).

The incubation period of human infection with ALSV is 3–7 days. The clinical symptoms include fever, persistent headache, fatigue, nausea, cervical lymphadenopathy, mild liver injury, and no death cases have been reported. ALSV can cause obvious cytopathic effect in Vero cells (Ren et al., 2025; Kholodilov et al., 2020; Zhang et al., 2020). Human infection is closely related to tick bites. The disease mainly affects field workers aged 40–60 years, and the cases are concentrated in May to July when ticks are most active. The virus infects livestock such as sheep and goats, and can also reproduce in tick cells, indicating a wide range of hosts (Kholodilov et al., 2020; Litov et al., 2024). The virus was detected in all life stages of ticks, including adults, nymphs and larvae. Vertical transmission is also recorded, allowing the virus to persist in the natural cycle (Ebert et al., 2023; Wu et al., 2023). In Northeast China and Russia, *Ixodes persulcatus* is the main vector, and *Ixodes ricinus* is dominant in Europe. ALSV is often co-infected with other Tick-Borne pathogens, which increases the difficulty of clinical diagnosis and treatment. At present, there is no vaccine or specific drug on the market. Most patients can recover within 6–8 days after symptomatic treatment (Ren et al., 2025; Wu et al., 2013; Casel et al., 2021).

Other emerging viruses, including Tacheng tick virus 1 and 2, have been detected in northwestern China and adjacent regions. Tacheng tick virus 1 was found in both *Dermacentor reticulatus* and *Ixodes ricinus*, providing the first evidence that *I. ricinus* may act as a competent vector (Ergunay et al., 2024b). Songling virus (SGLV) was isolated from patients with tick biting history in Heilongjiang Province, China. The virus has the typical genomic characteristics of the genus nemerovirus and forms an independent phylogenetic clade. SGLV can cause pathological changes in human liver cancer cells; It can cause febrile diseases, accompanied by headache, dizziness, discomfort and fatigue (Ma et al., 2021). Wetland virus (WELV) is a novel orthonairovirus first identified from a tick-bitten patient with fever and multiple organ dysfunction in Inner Mongolia, China, in 2019. It is closely related to the Hazara orthonairovirus genogroup, capable of inducing cytopathic effects in human cells and causing lethal infections in mice. WELV has been detected in 17 patients across northeastern China, ticks of five species, and multiple animals, with Haemaphysalis concinna possibly serving as its transovarial vector (Zhang et al., 2024b).

### 4.2 Novel Tick-Borne bacteria

Novel bacterial Tick-Borne pathogens are widespread and carry clear zoonotic risk. *Rickettsia barbariae* was first isolated from eggs of *Rhipicephalus turanicus* in northwestern China. Its genome carries the full set of virulence genes typical of spotted fever group rickettsiae and is closely related to the pathogenic *R. parkeri*, suggesting high disease potential (Wang et al., 2024). This pathogen is widely distributed and has been documented in Algeria, Israel, and Türkiye (Abdelkadir et al., 2019; Waner et al., 2014; Erol et al., 2025). Its vectors include ticks such as *Rhipicephalus turanicus, Hyalomma marginatum*, and *Rhipicephalus bursa*, while hosts also include the flea *Vermipsylla alakurt*, marbled polecats (*Vormela peregusna*), and domestic cats (Abdelkadir et al., 2019; Waner et al., 2014; Zhao et al., 2016; Liu et al., 2018; Erol et al., 2025). Although human infection has been confirmed, systematic data on its pathogenicity, infectivity, vector competence, and actual disease burden remain limited (Wang et al., 2024; Erol et al., 2025). A previously unrecognized rickettsia detected in ticks from Pakistan clusters phylogenetically with Asian *R. sibirica* and African *R. africae*, pointing to historical intercontinental transmission (Jamil et al., 2025).

*Anaplasma capra* has diversified into two genotypes across Asia, Europe, and Africa. Most human isolates belong to genotype 2, with a broad host range covering humans, domestic animals, and wildlife, and transmission by multiple hard tick species, making it a growing global zoonotic threat (Altay et al., 2024). Human infection presents with fever, headache, fatigue, rash and lymphadenopathy; severe cases show elevated hepatic transaminases (Li et al., 2015). Unlike other *Anaplasma* species, it exclusively infects erythrocytes (Peng et al., 2021a,b). Genotype 2 strains carry higher pathogenic potential and severe disease has been documented (Li et al., 2015). It is widely distributed in China, France, Spain, South Korea and Russia (Jouglin et al., 2025, 2022; Ullah et al., 2025; Altay et al., 2022; Remesar et al., 2022; Amer et al., 2019; Yang et al., 2017). Hosts include humans, goats, sheep, cattle, dogs, deer and rodents (Shi et al., 2019; Peng et al., 2018; Remesar et al., 2022; Amer et al., 2019; Yang et al., 2017; Miranda et al., 2021). Prevalence in goats ranges from 9%–78.6% in China and reaches 24% in France (Jouglin et al., 2022; Peng et al., 2018; Yang et al., 2017); in dogs it is 12.1% (Shi et al., 2019); in roe deer (Spain) 5.8% and in water deer (South Korea) 17.8% (Remesar et al., 2022; Amer et al., 2019). Confirmed vectors include *Haemaphysalis longicornis, Ixodes persulcatus, Rhipicephalus microplus, Dermacentor marginatus, Haemaphysalis concinna, Rhipicephalus turanicus* and *Alveonasus lahorensis* (Guo et al., 2019; Ullah et al., 2025; Yang et al., 2016; Tang et al., 2024; Tan et al., 2025). Pathogen detection in *H. concinna* salivary glands in France supports its vector competence (Jouglin et al., 2025). Tick infection rates are 1.32% (Hubei), 8%–63% (Shandong), 1.8% (Xinjiang) in China (Tang et al., 2024; Tan et al., 2025; Lu et al., 2023). Human cases are concentrated in East Asia and spreading to Europe, with a severe case rate of ~18% and fatal outcomes reported (Li et al., 2015). Coinfections with *Rickettsia, Borrelia* and *Anaplasma phagocytophilum* are common, exacerbating illness and complicating diagnosis (Guo et al., 2019; Miranda et al., 2021). Its prevalence and geographic range have expanded in recent years, making it an important emerging tick-borne zoonosis (Peng et al., 2018; Yang et al., 2017; Li et al., 2015). For comparative, the vector, geographical distribution and clinical characteristics of the key emerging Tick-Borne pathogens are summarized in Table 2.

**Table 2:** Vector, distribution, and clinical significance of key emerging Tick-Borne pathogens.

Pathogen	Vector	Distribution	Clinical significance
ALSV	*I. persulcatus, I. ricinus*	Asia, Europe	Fever, headache, fatigue, nausea, cervical lymphadenopathy, mild liver injury, no deaths
Tacheng tick virus	*D. reticulatus, I. ricinus*	NW China	Potential public health threat
SGLV	Ticks	Heilongjiang, China	Fever, headache, dizziness, fatigue
WELV	*H. concinna, etc*	NE China	Fever, organ dysfunction
*R. barbariae*	*R. turanicus, H. marginatum, R. sanguineus*	NW China, Algeria, Israel, Türkiye	Human infection confirmed
*A. capra*	Multiple hard ticks	China, France, Spain, South Korea, Russia	Fever, headache, fatigue, rash, lymphadenopathy; fatal cases reported

Data compiled from cited references.

### 4.3 Transmission dynamics and control of emerging Tick-Borne diseases

Climate warming and habitat alteration are driving the geographic expansion of tick vectors. These changes support the cross-regional transmission of diverse pathogens. Migratory birds and other mobile hosts further promote the long-distance dissemination of pathogens. For example, SFTSV has formed multicenter epidemic distributions through bird-mediated dispersal (Sheng et al., 2025). Both viral and bacterial pathogens improve environmental and host adaptability via genomic recombination and mutation. In tick-borne bunyaviruses, N-glycosylation of viral glycoproteins modulates virulence and host tropism (Shimojima et al., 2024). Interactions between the host skin microbiome and Tick-Borne pathogens have drawn growing research interest. Hamalin produced by *Akkermansia* can dampen inflammatory responses, offering new intervention targets against emerging infections (Xie et al., 2023).

Nearly all work in studies is limited to pathogen discovery and genome sequencing, lacking systematic assessments of their pathogenicity, infectivity, host range, vector competence, and human disease burden. Metagenomic studies mostly adopt convenience sampling methods and fail to cover the vast regions of Africa, South America, and tropical Asia. Research on microbiome interactions focuses only on single strains rather than community effects, which reduces the ecological realism of the studies. The development of diagnostic methods, vaccines, and drugs for these emerging pathogens has not yet been carried out, creating a critical gap in prevention and control preparedness.

### 4.4 Limitations and challenges

The lack of suitable animal models for viruses such as CCHF limits the evaluation of efficacy; The protective efficacy of SFTSV vaccines in the elder remains suboptimal, and the therapeutic efficacy of drugs in the later stages of the disease is poor; Lyme disease vaccines must strike a balance between immunogenicity and safety to avoid the risk of cross-reactivity (Wang et al., 2025; Zhang et al., 2025; Strnad et al., 2020). Pathogen genetic diversity and variation pose challenges to cross-protection provided by vaccines, necessitating the development of multivalent vaccines or the design of conservative antigens to address these issues (Chen et al., 2024b). In the future, there is a need to strengthen interdisciplinary collaboration, combining structural biology and immunomics to optimize antigen design, and utilizing novel delivery systems such as LNP to enhance immunogenicity; to advance multicentre clinical trials to clarify the efficacy and safety of drugs across different populations; and to strengthen cross-regional monitoring and collaboration under the principle of *One Health*, ultimately achieving precise and efficient prevention and control of tick-borne diseases.

Most vaccine candidates in studies do not take into account the genetic diversity of circulating strains, posing a risk of narrow protective spectrum. Clinical trials are small in scale, conducted in single centers, and have short follow-up periods, lacking real-world effectiveness data. Animal models often fail to accurately simulate human pathogenesis, reducing their predictive value.

## 5 New techniques for detection of Tick-Borne pathogens

The traditional detection technology of Tick-Borne pathogens generally has the defects of low detection flux and unable to identify unknown pathogens, which makes it difficult to achieve rapid and accurate detection. In recent years, CRISPR-Cas, metagenomic next-generation sequencing (mNGS) and microfluidics, have shown great potential in breaking through the above limitations, and have a good application prospect in the detection of Tick-Borne pathogens.

In the field of CRISPR technology, some researchers have constructed CRISPR/dCas9 biosensor, which can quickly distinguish scrub typhus and severe fever with thrombocytopenia syndrome (SFTS) within 20 min. The sensitivity is 100 times higher than that of RT-PCR, and can reach the single molecule level (Koo et al., 2018). For TBEV, RT-RPA combined with CRISPR/Cas13a and lateral flow dipstick can complete the detection within 1 h. The sensitivity and specificity of clinical samples are 100%, which is suitable for grass-roots site use (Zhang et al., 2024a). Similarly, the detection method based on RPA-CRISPR/Cas12a can detect *Anaplasma marginale* (4 copies/μL) and SFTSV (3 copies/reaction), which has strong specificity and is suitable for rapid detection in the field (Sutipatanasomboon et al., 2024; Huang et al., 2022). In addition, a one-step CRISPR/Cas12a detection system has been established to complete the detection of SFTSV within 45 min (Shu et al., 2025). In addition, RPA-CRISPR/Cas12a can also specifically detect *Ehrlichia canis* and *Anaplasma platys*, with higher sensitivity than PCR (Paenkaew et al., 2023). CRISPR/Cas12a combined with RT-RPA has also been used to rapidly detect multiple genotypes of SFTSV (Park et al., 2022). In addition, targeted retrieval was conducted for each novel diagnostic technology to supplement key original studies, and the backward and forward citation tracking methods were used to further identify relevant literature, ensuring no omission of important research.

Metagenomic next-generation sequencing has the advantages of broad-spectrum and unbiased detection, which can identify all known and unknown viruses, bacteria, fungi and parasites in the sample at one time, and has been widely used for the detection of Tick-Borne pathogens (Jiao et al., 2021).

According to the mNGS detection of *Dermacentor nuttalli* and *Ixodes persulcatus* in Inner Mongolia, China, *Candidatus Rickettsia tarasevichiae* was found in *Dermacentor nuttalli* in China for the first time (Jiao et al., 2021). Large scale mNGS monitoring of *Dermacentor* ticks in Mongolia detected *Rickettsia, Anaplasma, Bartonella*, etc., which clarified the high prevalence of tick-borne diseases in local livestock and provided a basis for cross-border transmission risk assessment (Altantogtokh et al., 2022). Shanghai, Qingdao and other places in China have found SGLV, Hubei tick virus and other new tick-borne viruses in *Haemaphysalis longicornis* and *Ixodes persulcatus* through mNGS, expanding the knowledge of tick viromics (Zeng et al., 2025; Hu et al., 2023).

In clinical application, mNGS has obvious advantages for laboratory diagnosis of atypical cases such as *Rickettsia*, TBEV, SFTSV, etc., which can quickly identify the pathogen, especially for cases without clear epidemiological exposure history (Zhang et al., 2023a). In patients with tick-borne encephalitis virus infection, the whole genome of the virus can be directly obtained through mNGS detection of cerebrospinal fluid samples, helping to trace the source of typing (Zakotnik et al., 2022). A 4-year-old encephalitis patient was diagnosed with Powassan virus (POWV) infection in Ohio, USA through mNGS (Farrington et al., 2023).

Although mNGS has obvious advantages, there are also challenges such as high cost, complex data analysis, insufficient sensitivity of detection of low abundance pathogens, and background microbial interference (Hu et al., 2023). In the future, with technological progress and cost reduction, mNGS will play an important role in the monitoring of Tick-Borne pathogens and early warning of new pathogens, providing important technical support for the prevention and control of tick-borne diseases. In addition, targeted retrieval was conducted for each novel diagnostic technology to supplement key original studies, and the backward and forward citation tracking methods were used to further identify relevant literature, ensuring no omission of important research.

Microfluidic is an important tool for the rapid detection of Tick-Borne pathogens by virtue of its advantages of miniaturization, high throughput and rapid detection, which can realize the synchronous detection of multiple samples and pathogens (Rodino and Pritt, 2021).

High-throughput microfluidic technology has been mature for large-scale epidemiological investigation of Tick-Borne pathogens. Ninety-six samples can be detected in a single run, covering a variety of Tick-Borne pathogens such as *Borrelia, Rickettsia*, and *Anaplasma*, significantly improving the detection efficiency (Moutailler and Galon, 2024). In the study of parasitic ticks on ruminants in Senegal, 36 kinds of pathogenic microorganisms can be screened at a time by using this technology (Rodino and Pritt, 2021). In the monitoring of ticks in tropical areas, microfluidic PCR combined with network analysis can simultaneously detect a variety of pathogens, revealing the co-infection mode and interaction relationship of pathogens (Díaz-Corona et al., 2024). The microfluidic chip based on RT-LAMP-CRISPR/Cas12b can realize portable and rapid detection of SFTS.The chip is driven by manual pressure without external power supply, and the detection limit is as low as 5 copies/reaction. The sensitivity of clinical samples is 88.9% and the specificity is 100% (Long et al., 2026).

With technological iterations, microfluidic chips will further integrate nucleic acid extraction, amplification and detection processes, and play a greater role in the large-scale monitoring of Tick-Borne pathogens (Boularias et al., 2021). In addition, targeted retrieval was conducted for each novel diagnostic technology to supplement key original studies, and the backward and forward citation tracking methods were used to further identify relevant literature, ensuring no omission of important research. In addition, targeted retrieval was conducted for each novel diagnostic technology to supplement key original studies, and the backward and forward citation tracking methods were used to further identify relevant literature, ensuring no omission of important research.

## 6 Conclusions and outlook

Ticks are major vectors of viral, bacterial, and protozoan pathogens that cause widespread zoonotic diseases, threatening human health and livestock production. Climate warming and human activity have expanded tick ranges and driven pathogen emergence, making tick-borne disease control an urgent global challenge. Recent advances in pathogen diversity, evolution, vector–pathogen–host interactions, control strategies, and countermeasure development have strengthened the evidence base for targeted intervention.

### 6.1 Priorities for future research

Future research must prioritize the following aspects:

1. Conduct sustained, global, and genetically informed surveillance of emerging Tick-Borne pathogens to improve relevant databases and risk maps;
2. Carry out mechanistic studies in natural vector-host systems to clarify the causal pathways of transmission and pathogenesis;
3. Develop broadly protective, thermostable, and age-optimized vaccines and therapeutics, focusing on the elderly and coinfected patients;
4. Build an integrated One Health system that organically combines ecological management, personal protection, surveillance and early warning, and cross-border collaboration.

### 6.2 Conclusion

In general, the research and prevention and control of Tick-Borne pathogens is a long-term and systematic project. It is necessary to face the limitations of existing research, abandon the research mode of single dimension and local area, and strengthen interdisciplinary, cross departmental and cross regional cooperation based on basic research, with technological innovation as the core and practical application as the guidance in the future, so as to continuously improve the scientific cognition level and comprehensive prevention and control ability of Tick-Borne diseases, minimize its harm to human health and animal husbandry development, and ensure public health safety and ecological environment stability.

## References

[B1] Abdelkadir K. Palomar A. Portillo A. Oteo J. Ait-Oudhia K. Khelef D. (2019). Presence of *Rickettsia aeschlimannii*, ‘Candidatus Rickettsia Barbariae' and coxiella burnetii in ticks from livestock in Northwestern Algeria. Ticks Tick Borne Dis. 10, 924–928. doi: 10.1016/j.ttbdis.2019.04.018 31080140

[B2] Akther S. Mongodin E. F. Morgan R. D. Di L. Yang X. Golovchenko M. . (2024). Natural selection and recombination at host-interacting lipoprotein loci drive genome diversification of lyme disease and related bacteria. MBio 15:e0174924. doi: 10.1128/mbio.01749-24 39145656 PMC11389397

[B3] Altantogtokh D. Lilak A. A. Takhampunya R. Sakolvaree J. Chanarat N. Matulis G. . (2022). Metagenomic profiles of dermacentor tick pathogens from across mongolia, using next generation sequencing. Front. Microbiol. 13:946631. doi: 10.3389/fmicb.2022.946631 36033893 PMC9399792

[B4] Altay K. Erol U. Sahin O. F. (2024). *Anaplasma capra*: a new emerging tick-borne zoonotic pathogen. Vet. Res. Commun. 48, 1329–1340. doi: 10.1007/s11259-024-10337-9 38424380 PMC11147849

[B5] Altay K. Erol U. Sahin O. F. Aytmirzakizi A. (2022). First molecular detection of anaplasma species in cattle from Kyrgyzstan; molecular identification of human pathogenic novel genotype *Anaplasma cap* and *Anaplasma phagocytophilum* related strain. Ticks Tick Borne Dis. 13:101861. doi: 10.1016/j.ttbdis.2021.101861 34773849

[B6] Amer S. Kim S. Yun Y. Na K.-J. (2019). Novel variants of the newly emerged *Anaplasma cap* from Korean water deer (hydropotes inermis argyropus) in South Korea. Parasit. Vectors 12:365. doi: 10.1186/s13071-019-3622-5 31345253 PMC6659236

[B7] Angulo F. J. Zhang P. Halsby K. Kelly P. Pilz A. Madhava H. . (2023). A systematic literature review of the effectiveness of tick-borne Encephalitis vaccines in Europe. Vaccine 41, 6914–6921. doi: 10.1016/j.vaccine.2023.10.014 37858450

[B8] Backus L. H. Foley J. E. Hobbs G. B. Bai Y. Beati L. (2022). A new species of tick, ixodes (ixodes) mojavensis (acari: Ixodidae), from the amargosa Valley of California. Ticks Tick Borne Dis. 13:102020. doi: 10.1016/j.ttbdis.2022.102020 35987116 PMC10917073

[B9] Bajer A. Dwużnik-Szarek D. (2021). The specificity of babesia-tick vector interactions: recent advances and pitfalls in molecular and field studies. Parasit. Vectors 14:507. doi: 10.1186/s13071-021-05019-3 34583754 PMC8480096

[B10] Baux E. Hansmann Y. Tranchant C. Roblot F. Arias P. Jaulhac B. . (2025). Guidelines for lyme borreliosis: clinical manifestations. Infect. Dis. Now. 55:105202. doi: 10.1016/j.idnow.2025.105202 41319868

[B11] Boulanger N. Boyer P. Talagrand-Reboul E. Hansmann Y. (2019). Ticks and tick-borne diseases. Med. Mal. Infect. 49, 87–97. doi: 10.1016/j.medmal.2019.01.007 30736991

[B12] Boularias G. Azzag N. Galon C. Šimo L. Boulouis H. Moutailler S. (2021). High-throughput microfluidic real-time pcr for the detection of multiple microorganisms in ixodid cattle ticks in Northeast Algeria. Pathogens 10, 362. doi: 10.3390/pathogens10030362 33803682 PMC8002991

[B13] Buczek A. M. Buczek W. Buczek A. Wysokinska-Miszczuk J. (2022). Food-borne transmission of tick-borne Encephalitis virus-spread, consequences, and prophylaxis. Int. J. Environ. Res. Public Health 19:1812. doi: 10.3390/ijerph19031812 35162837 PMC8835261

[B14] Casel M. A. Park S. J. Choi Y. K. (2021). Severe fever with thrombocytopenia syndrome virus: control strategy. Exp. Mol. Med. 53, 713–722. doi: 10.1038/s12276-021-00610-1 33953322 PMC8178303

[B15] Chen D. Lu Y. Wang W. Zhang Y. Liu T. Liu H. . (2024a). The prevalence of tick-borne encephalitis virus infection among humans in Heilongjiang Province of China in 2020-2023. Zoonoses Public Health 71, 955–961. doi: 10.1111/zph.13178 39169601

[B16] Chen X. Li Y. Wang X. (2024b). Multi-epitope vaccines: promising strategy against viral diseases in swine. Front. Cell. Infect. Microbiol. 14:1497580. doi: 10.3389/fcimb.2024.1497580 39760092 PMC11695243

[B17] Chen Z. Zhang J. Wang J. Tong H. Pan W. Ma F. . (2024c). N6-methyladenosine rna modification promotes severe fever with thrombocytopenia syndrome virus infection. PLoS Pathog. 20:e1012725. doi: 10.1371/journal.ppat.1012725 39585899 PMC11627400

[B18] Chiffi G. Grandgirard D. Leib S. L. Chrdle A. Ružek D. (2023). Tick-borne encephalitis: a comprehensive review of the epidemiology, virology, and clinical picture. Rev. Med. Virol. 33:e2470. doi: 10.1002/rmv.2470 37392370

[B19] Chitimia-Dobler L. Rieß R. Kahl O. Wölfel S. Dobler G. Nava S. . (2018). Ixodes inopinatus - occurring also outside the mediterranean region. Ticks Tick Borne Dis. 9, 196–200. doi: 10.1016/j.ttbdis.2017.09.004 28935402

[B20] Cialini C. Cafiso A. Waldeck M. Lundgren Å. Fält J. Settergren B. . (2025). Prevalence of tick-borne pathogens in feeding and questing ixodes ricinus ticks from southern sweden. Ticks Tick Borne Dis. 16:102453. doi: 10.1016/j.ttbdis.2025.102453 39946817

[B21] Couret J. Schofield S. Narasimhan S. (2022). The environment, the tick, and the pathogen - it is an ensemble. Front. Cell. Infect. Microbiol. 12:1049646. doi: 10.3389/fcimb.2022.1049646 36405964 PMC9666722

[B22] Cutler S. J. Vayssier-Taussat M. Estrada-Peña A. Potkonjak A. Mihalca A. D. Zeller H. (2021). Tick-borne diseases and co-infection: current considerations. Ticks Tick Borne Dis. 12:101607. doi: 10.1016/j.ttbdis.2020.101607 33220628

[B23] De la Fuente J. Estrada-Pena A. Venzal J. M. Kocan K. M. Sonenshine D. E. (2008). Overview: ticks as vectors of pathogens that cause disease in humans and animals. Front. Biosci. 13:3200. doi: 10.2741/3200 18508706

[B24] Díaz-Corona C. Roblejo-Arias L. Piloto-Sardiñas E. Díaz-Sánchez A. A. Foucault-Simonin A. Galon C. . (2024). Microfluidic pcr and network analysis reveals complex tick-borne pathogen interactions in the tropics. Parasit. Vectors 17:5. doi: 10.1186/s13071-023-06098-0 38178247 PMC10765916

[B25] Dong Q. Yang M. Li F. Jia Y. Rizabek K. Kairullayev K. . (2023). Spotted fever group *Rickettsiae* in hard ticks in Eastern and Southern Kazakhstan. Ticks Tick Borne Dis. 14;102238. doi: 10.1016/j.ttbdis.2023.102238 37722147

[B26] Drehmann M. Chitimia-Dobler L. Lindau A. Frank A. Mai S. Fachet K. . (2019). Ixodes frontalis: a neglected but ubiquitous tick species in Germany. Exp. Appl. Acarol. 78, 79–91. doi: 10.1007/s10493-019-00375-3 31093856

[B27] Du L. Shi W. Cui X. Fan H. Jiang J. Bian C. . (2025). Genome-resolved metagenomics reveals microbiome diversity across 48 tick species. Nat. Microbiol. 10, 2631–2645. doi: 10.1038/s41564-025-02119-z 40987851 PMC12488481

[B28] Du S. Wang Y. Wang J. Ma Y. Xu W. Shi X. . (2024). Ifitm3 inhibits severe fever with thrombocytopenia syndrome virus entry and interacts with viral gc protein. J. Med. Virol. 96:e29491. doi: 10.1002/jmv.29491 38402626

[B29] Ebert C. L. Söder L. Kubinski M. Glanz J. Gregersen E. Dümmer K. . (2023). Detection of alongshan virus in ticks and tick saliva from germany. Microorganisms 11:543. doi: 10.3390/microorganisms11030543 36985117 PMC10055853

[B30] Ergunay K. Boldbaatar B. Bourke B. P. Caicedo-Quiroga L. Tucker C. L. Letizia A. G. . (2024a). Metagenomic nanopore sequencing of tick-borne pathogens, Mongolia. Emerg. Infect. Dis. 30, 105–110. doi: 10.3201/eid3014.240128 PMC11559569 39530915

[B31] Ergunay K. Bourke B. P. Reinbold-Wasson D. D. Caicedo-Quiroga L. Vaydayko N. Kirkitadze G. . (2024b). Novel clades of tick-borne pathogenic nairoviruses in Europe. Infect. Genet. Evol. 121:105593. doi: 10.1016/j.meegid.2024.105593 38636618

[B32] Ergunay K. Golubiani G. Kirkitadze G. Reinbold-Wasson D. D. Bourke B. P. Phelps C. A. . (2025). Ongoing circulation of emerging tick-borne viruses in Poland, Eastern Europe. PLoS ONE 20:e0330544. doi: 10.1371/journal.pone.0330544 40901820 PMC12407414

[B33] Erol U. Sahin O. Urhan O. Genc M. Altay K. (2025). Primarily molecular detection and phylogenetic analyses of spotted fever group *Rickettsia* species in cats in Türkiye: with new host reports of *Rickettsia aeschlimannii, Rickettsia slovaca*, and *Candidatus rickettsia* barbariae. Comp. Immunol. Microbiol. Infect. Dis. 118:102319. doi: 10.1016/j.cimid.2025.102319 39923411

[B34] Fang L. Yu S. Tian X. Fu W. Su L. Chen Z. . (2023). Severe fever with thrombocytopenia syndrome virus replicates in platelets and enhances platelet activation. J. Thromb. Haemost. 21, 1336–1351. doi: 10.1016/j.jtha.2023.02.006 36792011

[B35] Farrington M. Elenz J. Ginsberg M. Chiu C. Miller S. Pangonis S. (2023). Powassan virus infection detected by metagenomic next-generation sequencing, Ohio, USA. Emerg. Infect. Dis. 29, 838–841. doi: 10.3201/eid2904.221005 36958034 PMC10045675

[B36] Fu B. Tang T. Yue M. Chen J. Su H. Huang X. . (2025). Mapping the global distribution of babesia infections. Transbound. Emerg. Dis. 2025:5889219. doi: 10.1155/tbed/5889219 41333613 PMC12668841

[B37] Ghavami M. B. Alibabaei Z. Jamavar M. R. Taghiloo B. (2024). Emergent spotted fever group *Rickettsiae* infections among hard ticks in Islamic Republic of Iran. East. Mediterr. Health J. 30, 145–155. doi: 10.26719/emhj.24.030 38491900

[B38] Gömer A. Lang A. Janshoff S. Steinmann J. Steinmann E. (2024). Epidemiology and global spread of emerging tick-borne alongshan virus. Emerg. Microbes Infect. 13:2404271. doi: 10.1080/22221751.2024.2404271 39259276 PMC11423535

[B39] Gray J. Kahl O. Zintl A. (2024). Pathogens transmitted by ixodes ricinus. Ticks Tick Borne Dis. 15:102402. doi: 10.1016/j.ttbdis.2024.102402 39368217

[B40] Guérin M. Shawky M. Zedan A. Octave S. Avalle B. Maffucci I. . (2023). Lyme borreliosis diagnosis: state of the art of improvements and innovations. BMC Microbiol. 23:204. doi: 10.1186/s12866-023-02935-5 37528399 PMC10392007

[B41] Guo W. Zhang B. Wang Y. Xu G. Wang X. Ni X. . (2019). Molecular identification and characterization of *Anaplasma cap* and *Anaplasma platys*-like in rhipicephalus microplus in Ankang, Northwest China. BMC Infect. Dis. 19:434. doi: 10.1186/s12879-019-4075-3 31101084 PMC6525361

[B42] Hu G. Jiang F. Luo Q. Zong K. Dong L. Mei G. . (2023). Diversity analysis of tick-borne viruses from hedgehogs and hares in Qingdao, China. Microbiol. Spectr. 11:e0534022. doi: 10.1128/spectrum.05340-22 37074196 PMC10269667

[B43] Huang M. Liu S. Xu Y. Li A. Wu W. Liang M. . (2022). Crispr/cas12a technology combined with rpa for rapid and portable sftsv detection. Front. Microbiol. 13:754995. doi: 10.3389/fmicb.2022.754995 35145502 PMC8822122

[B44] Jalovecka M. Malandrin L. Urbanova V. Mahmood S. Snebergerova P. Peklanska M. . (2024). Activation of the tick toll pathway to control infection of ixodes ricinus by the apicomplexan parasite *Babesia microti*. PLoS Pathog. 20:e1012743. doi: 10.1371/journal.ppat.1012743 39680508 PMC11649134

[B45] Jamil L. Li C. Wang Y. Jamil J. Tian W. Zhao D. . (2025). High diversity and low coinfections of pathogens in ticks from ruminants in Pakistan. Microorganisms 13:1276. doi: 10.3390/microorganisms13061276 40572164 PMC12195261

[B46] Jiao J. Lu Z. Yu Y. Ou Y. Fu M. Zhao Y. . (2021). Identification of tick-borne pathogens by metagenomic next-generation sequencing in dermacentor nuttalli and ixodes persulcatus in inner Mongolia, China. Parasit. Vectors 14:287. doi: 10.1186/s13071-021-04740-3 34044867 PMC8161991

[B47] Johnson N. Migné C. V. Gonzalez G. (2023). Tick-borne encephalitis. Curr. Opin. Infect. Dis. 36, 198–202. doi: 10.1097/QCO.0000000000000924 37093044

[B48] Jouglin M. Blanc B. Brunet A. Ortiz K. Malandrin L. (2025). *Anaplasma capra* and *Haemaphysalis concinna*: investigating a potential vector relationship in a wildlife reserve. Ticks Tick Borne Dis. 16:102500. doi: 10.1016/j.ttbdis.2025.102500 40483925

[B49] Jouglin M. Rispe C. Grech-Angelini S. Gallois M. Malandrin L. (2022). *Anaplasma cap* in sheep and goats on corsica island, france: a European lineage within a cap clade ii? Ticks Tick Borne Dis 13:101934. doi: 10.1016/j.ttbdis.2022.101934 35263704

[B50] Karshima S. N. Ahmed M. I. Kogi C. A. Iliya P. S. (2022). *Anaplasma phagocytophilum* infection rates in questing and host-attached ticks: a global systematic review and meta-analysis. Acta Trop. 228:106299. doi: 10.1016/j.actatropica.2021.106299 34998998

[B51] Kholodilov I. S. Litov A. G. Klimentov A. S. Belova O. A. Polienko A. E. Nikitin N. A. . (2020). Isolation and characterisation of alongshan virus in Russia. Viruses 12:362. doi: 10.3390/v12040362 32224888 PMC7232203

[B52] Kim H. K. (2022). *Rickettsia*-host-tick interactions: knowledge advances and gaps. Infect. Immun. 90:e0062121. doi: 10.1128/iai.00621-21 35993770 PMC9476906

[B53] Koo B. Kim D. Kweon J. Jin C. E. Kim S. Kim Y. . (2018). Crispr/dcas9-mediated biosensor for detection of tick-borne diseases. Sensors Actuat. B: Chem. 273, 316–321. doi: 10.1016/j.snb.2018.06.069 32288252 PMC7126152

[B54] Laredo-Tiscareño S. V. Garza-Hernandez J. A. Tangudu C. S. Dankaona W. Rodríguez-Alarc´on C. A. Gonzalez-Peña R. . (2025). Detection of multiple novel viruses in argasid and ixodid ticks in Mexico. Ticks Tick Borne Dis. 16:102455. doi: 10.1016/j.ttbdis.2025.102455 39946816

[B55] Li H. Zheng Y. Ma L. Jia N. Jiang B. Jiang R. . (2015). Human infection with a novel tick-borne anaplasma species in china: a surveillance study. Lancet Infect. Dis. 15, 663–670. doi: 10.1016/S1473-3099(15)70051-4 25833289

[B56] Li P. Hui S. Chong Z. Escaffre O. Nguyen M. N. Muraro S. P. . (2025). Lrp8 is an entry receptor for tick-borne encephalitis viruses. Proc. Natl. Acad. Sci. U. S. A. 122:e2525771122. doi: 10.1073/pnas.2525771122 41166431 PMC12595491

[B57] Ličková M. Fumačová H. S. Sláviková M. Klempa B. (2021). Alimentary infections by tick-borne Encephalitis virus. Viruses 14:56. doi: 10.3390/v14010056 35062261 PMC8779402

[B58] Litov A. G. Kholodilov I. S. Belova O. A. Gadzhikurbanov M. N. Ivannikova A. Y. Karganova G. G. . (2024). High-throughput sequencing reveals three rhabdoviruses persisting in the ire/ctvm19 cell line. Viruses 16:576. doi: 10.3390/v16040576 38675918 PMC11054507

[B59] Liu X. Yang M. Liu G. Zhao S. Yuan W. Xiao R. . (2018). Molecular evidence of *Rickettsia raoultii*, “Candidatus Rickettsia Barbariae” and a novel babesia genotype in marbled polecats (*Vormela peregusna*) at the China-Kazakhstan border. Paras. Vect. 11:450. doi: 10.1186/s13071-018-3033-z 30075738 PMC6090811

[B60] Long Y. Sun S. Mei H. Zhou D. Zhou H. Fang Z. . (2026). Rt-lamp-crispr/cas12b-based hand-pressure-actuated microfluidic chip for rapid and portable detection of severe fever with thrombocytopenia syndrome virus. Talanta 301:129277. doi: 10.1016/j.talanta.2025.129277 41448060

[B61] Lu M. Meng C. Li Y. Zhou G. Wang L. Xu X. . (2023). *Rickettsia* sp. and *Anaplasma* spp. in haemaphysalis longicornis from Shandong Province of China. Ticks Tick Borne Dis 14:102082. doi: 10.1016/j.ttbdis.2022.102082 36403321

[B62] Ma J. Lv X. Zhang X. Han S. Wang Z. Li L. . (2021). Identification of a new orthonairovirus associated with human febrile illness in China. Nat. Med. 27, 434–439. doi: 10.1038/s41591-020-01228-y 33603240

[B63] Ma W. Gao L. Wu X. Zhong L. Huang X. Yang R. . (2024). Global prevalence of *Borrelia burgdorferi* and *Anaplasma phagocytophilum* coinfection in wild and domesticated animals: a systematic review and meta-analysis. J. Glob. Health 14:4231. doi: 10.7189/jogh.14.04231 39641312 PMC11622344

[B64] Marques A. R. Strle F. Wormser G. P. (2021). Comparison of lyme disease in the United States and Europe. Emerg. Infect. Dis. 27, 2017–2024. doi: 10.3201/eid2708.204763 34286689 PMC8314816

[B65] Martiniano N. O. d. M. Sato T. P. Vizzoni V. F. Ventura S. d. F. Oliveira S. V. d. Amorim M. . (2022). A new focus of spotted fever caused by *Rickettsia* Parkeri in Brazil. Rev. Inst. Med. Trop. São Paulo 64:e22. doi: 10.1590/s1678-9946202264022 35293560 PMC8916588

[B66] Mead P. (2022). Epidemiology of lyme disease. Infect. Dis. Clin. North Am. 36, 495–521. doi: 10.1016/j.idc.2022.03.004 36116831

[B67] Miranda E. A. Han S.-W. Cho Y.-K. Choi K.-S. Chae J.-S. (2021). Co-infection with *Anaplasma* species and novel genetic variants detected in cattle and goats in the Republic of Korea. Pathogens 10:28. doi: 10.3390/pathogens10010028 33401478 PMC7830860

[B68] Moutailler S. Galon C. (2024). Real-time microfluidic pcrs: a high-throughput method to detect 48 or 96 tick-borne pathogens. Methods Mol. Biol. 2742, 1–17. doi: 10.1007/978-1-0716-3561-2_1 38165611

[B69] Narvaez Z. E. Egizi A. M. Price D. C. (2024). First record of ixodes Keiransi (acari: Ixodidae) in New Jersey, USA. J. Med. Entomol. 61, 798–801. doi: 10.1093/jme/tjae037 38493309

[B70] Ni X. Cui X. Liu J. Ye R. Wu Y. Jiang J. . (2023). Metavirome of 31 tick species provides a compendium of 1,801 rna virus genomes. Nat. Microbiol. 8, 162–173. doi: 10.1038/s41564-022-01275-w 36604510 PMC9816062

[B71] Ning T. Qiang C. Chao-Pin L. (2017). [Ixodes ovatus found in Huainan area in Anhui Province]. Chin. J. Schistosomiasis Control. 29:647 (In Chinese). doi: 10.16250/j.32.1374.2016173 29469370

[B72] O'Bier N. S. Camire A. C. Patel D. T. Billingsley J. S. Hodges K. R. Marconi R. T. . (2024). Development of novel multi-protein chimeric immunogens that protect against infection with the lyme disease agent, *Borreliella burgdorferi*. MBio 15:e0215924. doi: 10.1128/mbio.02159-24 39287439 PMC11481559

[B73] Paenkaew S. Jaito N. Pradit W. Chomdej S. Nganvongpanit K. Siengdee P. . (2023). Rpa/crispr-cas12a as a specific, sensitive and rapid method for diagnosing *Ehrlichia canis* and *Anaplasma platys* in dogs in Thailand. Vet. Res. Commun. 47, 1601–1613. doi: 10.1007/s11259-023-10114-0 36997812 PMC10062689

[B74] Parfut A. Laugel E. Baer S. Gonzalez G. Hansmann Y. Wendling M. . (2023). Tick-borne encephalitis in pediatrics: an often overlooked diagnosis. Infect. Dis. Now. 53:104645. doi: 10.1016/j.idnow.2023.01.005 36642097

[B75] Park B. Yoo J. Heo S. Kim M. Lee K. Song Y. (2022). A crispr-cas12a-based diagnostic method for multiple genotypes of severe fever with thrombocytopenia syndrome virus. PLoS Negl. Trop. Dis. 16:e0010666. doi: 10.1371/journal.pntd.0010666 35917293 PMC9345333

[B76] Park J. M. Genera B. M. Fahy D. Swallow K. T. Nelson C. M. Oliver J. D. . (2023). An *Anaplasma phagocytophilum* t4ss effector, atea, is essential for tick infection. MBio 14:e0171123. doi: 10.1128/mbio.01711-23 37747883 PMC10653876

[B77] Peng Y. Lu C. Yan Y. Shi K. Chen Q. Zhao C. . (2021a). The first detection of *Anaplasma cap*, an emerging zoonotic *Anaplasma* sp., in erythrocytes. Emerg. Microbes Infect. 10, 226–234. doi: 10.1080/22221751.2021.1876532 33446064 PMC7894429

[B78] Peng Y. Lu C. Yan Y. Song J. Pei Z. Gong P. . (2021b). The novel zoonotic pathogen, anaplasma cap, infects human erythrocytes, hl-60, and tf-1 cells *in vitro*. Pathogens 10:600. doi: 10.3390/pathogens10050600 34069112 PMC8156996

[B79] Peng Y. Wang K. Zhao S. Yan Y. Wang H. Jing J. . (2018). Detection and phylogenetic characterization of anaplasma cap: an emerging pathogen in sheep and goats in china. Front. Cell. Infect. Microbiol. 8:283. doi: 10.3389/fcimb.2018.00283 30214896 PMC6126426

[B80] Pfeifle A. Anderson-Duvall R. Tamming L. A. Zhang W. Thulasi R. S. N. Gravel C. . (2025). Borrelia burgdorferi strain-specific differences in mouse infectivity and pathology. Pathogens 14:352. doi: 10.3390/pathogens14040352 40333117 PMC12029986

[B81] Price L. E. Winter J. M. Cantoni J. L. Cozens D. W. Linske M. A. Williams S. C. . (2024). Spatial and temporal distribution of ixodes scapularis and tick-borne pathogens across the Northeastern United States. Parasit. Vect. 17:481. doi: 10.1186/s13071-024-06518-9 39574137 PMC11583392

[B82] Pulkkinen L. I. A. Barrass S. V. Domanska A. Överby A. K. Anastasina M. Butcher S. J. (2022). Molecular organisation of tick-borne encephalitis virus. Viruses 14:792. doi: 10.3390/v14040792 35458522 PMC9027435

[B83] Radolf J. D. Strle K. Lemieux J. E. Strle F. (2021). Lyme disease in humans. Curr. Issues Mol. Biol. 42, 333–384. doi: 10.21775/cimb.042.333 33303701 PMC7946767

[B84] Rana V. S. Kitsou C. Dumler J. S. Pal U. (2023). Immune evasion strategies of major tick-transmitted bacterial pathogens. Trends Microbiol. 31, 62–75. doi: 10.1016/j.tim.2022.08.002 36055896 PMC9772108

[B85] Remesar S. Prieto A. García-Dios D. López-Lorenzo G. Martínez-Calabuig N. Díaz-Cao J. M. . (2022). Diversity of anaplasma species and importance of mixed infections in roe deer from Spain. Transbound. Emerg. Dis. 69, e374–e385. doi: 10.1111/tbed.14319 34529897

[B86] Ren M. Pang Z. Tu Y. Wang A. Xu T. Yu X. . (2025). Alongshan virus: an emerging arboviral challenge in regional health security. Virulence 16:2492360. doi: 10.1080/21505594.2025.2492360 40233926 PMC12001551

[B87] Rodino K. Pritt B. (2021). Novel applications of metagenomics for detection of tickborne pathogens. Clin. Chem. 68, 69–74. doi: 10.1093/clinchem/hvab228 34969117

[B88] Rodrigues A. C. C. M. B. Labruna M. B. Szabó M. P. J. (2023). The inoculation eschar of *Rickettsia parkeri* rickettsiosis in brazil: importance and cautions. Ticks Tick Borne Dis. 14:102127. doi: 10.1016/j.ttbdis.2023.102127 36693294

[B89] Rosendal E. Lindqvist R. Chotiwan N. Henriksson J. överby A. K. (2024). Transcriptional response to tick-borne flavivirus infection in neurons, astrocytes and microglia *in vivo* and *in vitro*. Viruses 16:1327. doi: 10.3390/v16081327 39205301 PMC11359927

[B90] Samaddar S. Rolandelli A. O'Neal A. J. Laukaitis-Yousey H. J. Marnin L. Singh N. . (2024). Bacterial reprogramming of tick metabolism impacts vector fitness and susceptibility to infection. Nat. Microbiol. 9, 2278–2291. doi: 10.1038/s41564-024-01756-0 38997520 PMC11926704

[B91] Seo J. Kim D. Yun N. Kim D. (2021). Clinical update of severe fever with thrombocytopenia syndrome. Viruses 13:1213. doi: 10.3390/v13071213 34201811 PMC8310018

[B92] Sheng R. Cheng T. Wang Y. Wen H. Swanstrom R. (2025). Molecular evolution and geographic migration of severe fever with thrombocytopenia syndrome virus in Asia. PLoS Pathog. 21:e1012970. doi: 10.1371/journal.ppat.1012970 40053514

[B93] Shi K. Li J. Yan Y. Chen Q. Wang K. Zhou Y. . (2019). Dogs as new hosts for the emerging zoonotic pathogen anaplasma cap in china. Front. Cell. Infect. Microbiol. 9:394. doi: 10.3389/fcimb.2019.00394 31850236 PMC6901931

[B94] Shimojima M. Sugimoto S. Taniguchi S. Maeki T. Yoshikawa T. Kurosu T. . (2024). N-glycosylation of viral glycoprotein is a novel determinant for the tropism and virulence of highly pathogenic tick-borne bunyaviruses. PLoS Pathog. 20:e1012348. doi: 10.1371/journal.ppat.1012348 39008518 PMC11271937

[B95] Shu J. Tan Q. Huang Z. Zhang T. Ye L. Fu S. . (2025). One-pot one-step detection platform for severe fever with thrombocytopenia syndrome virus via the crispr/cas12a detection system. Virol. J. 22:203. doi: 10.1186/s12985-025-02772-0 40551124 PMC12186318

[B96] Socarras K. M. Haslund-Gourley B. S. Cramer N. A. Comunale M. A. Marconi R. T. Ehrlich G. D. (2022). Large-scale sequencing of borreliaceae for the construction of pan-genomic-based diagnostics. Genes (Basel) 13:1604. doi: 10.3390/genes13091604 36140772 PMC9498496

[B97] Strausz S. Abner E. Blacker G. Galloway S. Hansen P. Feng Q. . (2024). Scgb1d2 inhibits growth of borrelia burgdorferi and affects susceptibility to lyme disease. Nat. Commun. 15:2041. doi: 10.1038/s41467-024-45983-9 38503741 PMC10950847

[B98] Strnad M. Grubhoffer L. Rego R. O. M. (2020). Novel targets and strategies to combat borreliosis. Appl. Microbiol. Biotechnol. 104, 1915–1925. doi: 10.1007/s00253-020-10375-8 31953560 PMC7222997

[B99] Sui L. Wang W. Guo X. Zhao Y. Tian T. Zhang J. . (2024). Multi-protomics analysis identified host cellular pathways perturbed by tick-borne encephalitis virus infection. Nat. Commun. 15:10435. doi: 10.1038/s41467-024-54628-w 39616195 PMC11608235

[B100] Sun H. Hu Q. Lu S. Yang Y. Zhang L. Long J. . (2025). Current status of severe fever with thrombocytopenia syndrome in China (review). Int. J. Mol. Med. 56:169. doi: 10.3892/ijmm.2025.5610 40849814 PMC12373438

[B101] Sutipatanasomboon A. Wongsantichon J. Sakdee S. Naksith P. Watthanadirek A. Anuracpreeda P. . (2024). Rpa-crispr/cas12a assay for the diagnosis of bovine anaplasma marginale infection. Sci. Rep. 14:7820. doi: 10.1038/s41598-024-58169-6 38570576 PMC10991388

[B102] Szczotko M. Antunes S. Domingos A. Kubiak K. Dmitryjuk M. (2024). Tick-borne pathogens and defensin genes expression: a closer look at ixodes ricinus and dermacentor reticulatus. Dev. Comp. Immunol. 160:105231. doi: 10.1016/j.dci.2024.105231 39043336

[B103] Sze C. W. Lynch M. J. Zhang K. Neau D. B. Ealick S. E. Crane B. R. . (2025). Lactate dehydrogenase is the Achilles' heel of lyme disease bacterium *Borreliella burgdorferi*. MBio 16:e0372824. doi: 10.1128/mbio.03728-24 40111021 PMC11980376

[B104] Tan H. Dong X. Kang J. Bu N. Zhang Y. Qi Z. . (2025). Molecular characterization of livestock-associated ticks and tick-borne bacteria in Xinjiang, Northwestern China. Parasit. Vect. 19:12. doi: 10.1186/s13071-025-07178-z 41331487 PMC12777473

[B105] Tang J. Xu J. Liu X. Lv F. Yao Q. Zhou X. . (2024). Prevalence and genetic diversity of *Anaplasma* and *Ehrlichia* in ticks and domesticated animals in Suizhou County, Hubei Province, china. Sci. Rep. 14:12621. doi: 10.1038/s41598-024-63267-6 38824201 PMC11144266

[B106] Taylor-Salmon E. Shapiro E. D. (2024). Tick-borne infections in children in North America. Curr. Opin. Pediatr. 36, 156–163. doi: 10.1097/MOP.0000000000001326 38167816 PMC10932821

[B107] Tian D. Ye R. Li Y. Wang N. Gao W. Wang B. . (2025). Virome specific to tick genus with distinct ecogeographical distribution. Microbiome 13:57. doi: 10.1186/s40168-025-02061-6 40022268 PMC11869668

[B108] Ullah S. Khan A. Cossío-Bayúgar R. Giantsis I. A. Niaz S. Nasreen N. . (2025). Molecular detection of anaplasma cap and anaplasma marginale in rhipicephalus microplus ticks infesting cows. Sci. Rep. 15:34388. doi: 10.1038/s41598-025-17263-z 41038925 PMC12491612

[B109] Veinović G. Sukara R. Mihaljica D. Penezić A. Ćirović D. Tomanovic S. (2024). The occurrence and diversity of tick-borne pathogens in small mammals from Serbia. Vector Borne Zoonotic Dis. 24, 285–292. doi: 10.1089/vbz.2023.0088 38346321

[B110] Waner T. Keysary A. Eremeeva M. E. Din A. B. Mumcuoglu K. Y. King R. . (2014). *Rickettsia africae* and *Candidatus rickettsia* barbariae in ticks in Israel. Am. J. Trop. Med. Hyg. 90, 920–922. doi: 10.4269/ajtmh.13-0697 24615133 PMC4015588

[B111] Wang N. Yu H. Han X. Li C. Ye R. Du L. . (2024). Genomic characterization of an emerging *Rickettsia barbariae* isolated from tick eggs in Northwestern China. Emerg. Microbes Infect. 13:2396870. doi: 10.1080/22221751.2024.2396870 39193640 PMC11378659

[B112] Wang X. Zheng X. Ge H. Cui N. Lin L. Yue M. . (2025). Metformin as antiviral therapy protects hyperglycemic and diabetic patients. MBio 16:e0063425. doi: 10.1128/mbio.00634-25 40391966 PMC12153287

[B113] Wen Y. Ni Z. Hu Y. Wu J. Fang Y. Zhang G. . (2024). Multiple genotypes and reassortants of severe fever with thrombocytopenia syndrome virus co-circulating in Hangzhou in Southeastern China, 2013-2023. J. Med. Virol. 96:e70029. doi: 10.1002/jmv.70029 39530174

[B114] Wimms C. Aljundi E. Halsey S. J. (2023). Regional dynamics of tick vectors of human disease. Curr. Opin. Insect. Sci. 55:101006. doi: 10.1016/j.cois.2023.101006 36702303

[B115] Woo D. Michelow I. C. Choi Y. Lee H. Park S. (2025). Transmission of severe fever with thrombocytopenia syndrome (sfts) to humans: a systematic review of individual participant data and meta-analysis. J. Infect. Public Health 18:102685. doi: 10.1016/j.jiph.2025.102685 40073663

[B116] Wu X. Na R. Wei S. Zhu J. Peng H. (2013). Distribution of tick-borne diseases in china. Parasit. Vect. 6:119. doi: 10.1186/1756-3305-6-119 23617899 PMC3640964

[B117] Wu Z. Chen J. Zhang L. Zhang Y. Liu L. Niu G. (2023). Molecular evidence for transovarial transmission of Jingmen tick virus. J. Med. Virol. 95:e28357. doi: 10.1002/jmv.28357 36443647

[B118] Xie J. Li H. Zhang X. Yang T. Yue M. Zhang Y. . (2023). *Akkermansia muciniphila* protects mice against an emerging tick-borne viral pathogen. Nat. Microbiol. 8, 91–106. doi: 10.1038/s41564-022-01279-6 36604506

[B119] Yang J. Liu Z. Niu Q. Liu J. Han R. Guan G. . (2017). A novel zoonotic anaplasma species is prevalent in small ruminants: potential public health implications. Parasit. Vect. 10:264. doi: 10.1186/s13071-017-2182-9 28558749 PMC5450374

[B120] Yang J. Liu Z. Niu Q. Liu J. Han R. Liu G. . (2016). Molecular survey and characterization of a novel *Anaplasma* species closely related to anaplasma cap in ticks, Northwestern China. Parasit. Vect. 9:603. doi: 10.1186/s13071-016-1886-6 PMC5123347 27884197

[B121] Yu X. Jiang C. Jia S. Chang S. Li Y. Liu L. . (2026). Favipiravir's clinical potential for treating severe fever with thrombocytopenia syndrome (sfts): a narrative review. Virology 617:110799. doi: 10.1016/j.virol.2026.110799 41587499

[B122] Yuan F. Zhu L. Tian D. Xia M. Zheng M. Zhang Q. . (2024). The first discovery of severe fever with thrombocytopenia virus in the center of Metropolitan Beijing, China. Virol. Sin. 39, 875–881. doi: 10.1016/j.virs.2024.11.002 39522880 PMC11738777

[B123] Zakotnik S. Knap N. Bogovič P. Zorec T. M. Poljak M. Strle F. . (2022). Complete genome sequencing of tick-borne encephalitis virus directly from clinical samples: comparison of shotgun metagenomic and targeted amplicon-based sequencing. Viruses 14:1267. doi: 10.3390/v14061267 35746738 PMC9231111

[B124] Zeng W. Yang L. Cui L. Liang C. Zhu D. Fang Y. . (2025). Virome analysis and detection of ticks and tick-borne viruses in Shanghai, China. Front. Microbiol. 16:1699705. doi: 10.3389/fmicb.2025.1699705 41199942 PMC12586129

[B125] Zhang H. Wang Y. Chen C. Xing W. Xia W. Fu W. . (2024a). A novel rapid visual nucleic acid detection technique for tick-borne encephalitis virus by combining rt-recombinase-aided amplification and crispr/cas13a coupled with a lateral flow dipstick. Int. J. Biol. Macromol. 275:133720. doi: 10.1016/j.ijbiomac.2024.133720 38987000

[B126] Zhang S. Shang H. Han S. Li J. Peng X. Wu Y. . (2025). Discovery and characterization of potent broadly neutralizing antibodies from human survivors of severe fever with thrombocytopenia syndrome. EBioMedicine 111:105481. doi: 10.1016/j.ebiom.2024.105481 39644769 PMC11665701

[B127] Zhang X. Chen H. Han D. Wu W. (2023a). Clinical usefulness of metagenomic next-generation sequencing for rickettsia and *Coxiella burnetii* diagnosis. Eur. J. Clin. Microbiol. Infect. Dis. 42, 681–689. doi: 10.1007/s10096-023-04586-w 36997767 PMC10172222

[B128] Zhang X. Wang N. Wang Z. Liu Q. (2020). The discovery of segmented flaviviruses: implications for viral emergence. Curr. Opin. Virol. 40, 11–18. doi: 10.1016/j.coviro.2020.02.001 32217446

[B129] Zhang X. A. Ma Y. D. Zhang Y. F. Hu Z. Y. Zhang J. T. Han S. . (2024b). A new orthonairovirus associated with human febrile illness. N. Engl. J. Med. 391, 821–831. doi: 10.1056/NEJMoa2313722 39231344

[B130] Zhang Y. Y. Sun Y. Q. Chen J. J. Teng A. Y. Wang T. Li T . (2023b). Mapping the global distribution of spotted fever group rickettsiae: a systematic review with modelling analysis. Lancet Digit Health 5, e5–e15. doi: 10.1016/S2589-7500(22)00212-6 36424337 PMC10039616

[B131] Zhao S. Li H. Yin X. Liu Z. Chen C. Wang Y. (2016). First detection of *Candidatus rickettsia barbariae* in the flea vermipsylla alakurt from North-Western China. Paras. Vect. 9:325. doi: 10.1186/s13071-016-1614-2 27267467 PMC4895814

[B132] Zhao Y. Sui L. Pan M. Jin F. Huang Y. Fang S. . (2024). Alongshan virus degrades stat2 to suppress innate immunity. J. Virol. 99:e0130124. doi: 10.1128/jvi.01301-24 39655955 PMC11784234

[B133] Zhu W. Ye R. Tian D. Wang N. Gao W. Wang B. . (2025). The first direct detection of spotted fever group *Rickettsia* spp. diversity in ticks from Ningxia, Northwestern China. PLoS Negl. Trop. Dis. 19:e0012729. doi: 10.1371/journal.pntd.0012729 39746018 PMC11695002

[B134] Zintl A. McManus A. Galan M. Diquattro M. Giuffredi L. Charbonnel N. . (2023). Presence and identity of babesia microti in Ireland. Ticks Tick Borne Dis. 14:102221. doi: 10.1016/j.ttbdis.2023.102221 37406478

